# Hyperleptinemia in Neonatally Overfed Female Rats Does Not Dysregulate Feeding Circuitry

**DOI:** 10.3389/fendo.2017.00287

**Published:** 2017-10-25

**Authors:** Ilvana Ziko, Luba Sominsky, Thai-Xinh Nguyen, Kit-Yi Yam, Simone De Luca, Aniko Korosi, Sarah J. Spencer

**Affiliations:** ^1^School of Health and Biomedical Sciences RMIT University, Melbourne, VIC, Australia; ^2^Swammerdam Institute for Life Sciences, Center for Neuroscience, University of Amsterdam, Amsterdam, Netherlands

**Keywords:** hypothalamus, nutrition, leptin, sex, satiety, obesity, neonatal

## Abstract

Neonatal overfeeding during the first weeks of life in male rats is associated with a disruption in the peripheral and central leptin systems. Neonatally overfed male rats have increased circulating leptin in the first 2 weeks of life, which corresponds to an increase in body weight compared to normally fed counterparts. These effects are associated with a short-term disruption in the connectivity of neuropeptide Y (NPY), agouti-related peptide (AgRP), and pro-opiomelanocortin (POMC) neurons within the regions of the hypothalamus responsible for control of energy balance and food intake. Female rats that are overfed during the first weeks of their life experience similar changes in circulating leptin levels as well as in their body weight. However, it has not yet been studied whether these metabolic changes are associated with the same central effects as observed in males. Here, we hypothesized that hyperleptinemia associated with neonatal overfeeding would lead to changes in central feeding circuitry in females as it does in males. We assessed hypothalamic NPY, AgRP, and POMC gene expression and immunoreactivity at 7, 12, or 14 days of age, as well as neuronal activation in response to exogenous leptin in neonatally overfed and control female rats. Neonatally overfed female rats were hyperleptinemic and were heavier than controls. However, these metabolic changes were not mirrored centrally by changes in hypothalamic NPY, AGRP, and POMC fiber density. These findings are suggestive of sex differences in the effects of neonatal overfeeding and of differences in the ability of the female and male central systems to respond to changes in the early life nutritional environment.

## Introduction

Early life obesity is associated with increased risk of developing diabetes, cardiovascular complications and, consequently, increased rates of premature death (1, 2). Clinical studies have shown an association between the nutritional environment during early life and obesity, and obesity-related comorbidities, in adulthood (3). Similarly, in a neonatally overfed animal model representative of childhood obesity, both male and female rat pups raised in small litters, with greater access to their mothers’ milk, experience significantly increased body weight, accompanied by obesity-related comorbidities in adulthood compared to pups raised in normal litters. These comorbidities include hypothalamic–pituitary–adrenal (HPA) axis dysfunction, impaired reproductive function, memory deficits, and many other effects (4–6). These neonatally overfed rats are also hyperleptinemic throughout life (7–12) and males, at least, are leptin resistant in the neonatal period (13–16).

In healthy adults, leptin, one of the main central regulators of energy balance and satiety, acts by activating hypothalamic pro-opiomelanocortin (POMC) and cocaine and amphetamine-regulated transcript anorexigenic neurons and inhibiting neuropeptide Y (NPY) and agouti-related peptide (AgRP) orexigenic neurons to suppress feeding (17, 18). Recent data have shown, at least in males, an additional important role of leptin in the development of hypothalamic pathways that are related to feeding (19, 20). In rodents, a key developmental window for hypothalamic feeding circuitry occurs around postnatal day (P) 4–16. This period coincides with naturally occurring high levels of leptin (20, 21), and this elevated leptin stimulates the growth of connections between the arcuate nucleus of the hypothalamus (ARC), paraventricular nucleus of the hypothalamus (PVN), dorsomedial hypothalamus, and lateral hypothalamus, a process that may later be curtailed by ghrelin (20, 22, 23). In neonates, in the absence of leptin or ghrelin, or presence of a premature leptin surge or delayed ghrelin rise, neuronal connections between the relevant hypothalamic brain regions are impaired, leading to metabolic complications and obesity long term (20, 23, 24).

Elevated circulating leptin from as early as P7, indicative of a premature leptin surge, can be seen with neonatal overfeeding (12, 14). As such, early life overnutrition in male mice leads to hyperleptinemia, and this is associated with early leptin resistance, accelerated weight gain, and increased fat mass in comparison to controls (25). These mice also have disturbances in the ghrelin system, including reduced central responses to exogenous ghrelin (26). Similarly, neonatal overfeeding in male rats leads to hyperleptinemia and suppressed circulating ghrelin, and this is associated with a dysregulation in NPY and AgRP neuronal networks within the ARC and PVN (12, 13, 27). As shown in our previous work, neonatal overfeeding strongly increases the density of NPY and AgRP neuronal fibers in the ARC, while reducing the density of such fibers in the PVN, compared to normally fed controls (13). This profile is associated with reduced hypothalamic sensitivity to exogenous leptin and increased sensitivity to ghrelin, at least in the neonatal phase (13, 14, 27). However, at least some of these effects may be specific to males (4).

Neonatal overfeeding leads to weight gain, exacerbated pro-inflammatory responses to immune challenge, and microglial priming in female rodents that are very similar to the effects seen in males (28–30). However, there are some noteworthy differences between the sexes. We have previously seen that neonatal overfeeding in females is associated with unique changes in the female HPA axis such that females, but not males, have exacerbated PVN responses to psychological stress accompanied by increased open arm exploration in the elevated plus maze, potentially related to an impaired ability of the female pituitary gland to respond to ghrelin (4, 10). Males, but not females, that are neonatally overfed also have disruptions in both circulating ghrelin and the ability of ghrelin to act at the hypothalamus to regulate feeding circuitry (4, 27); an effect that is present during the neonatal period but largely normalized by adulthood. Like males, neonatally overfed females are hyperleptinemic in juvenile and adult life (5, 12). However, it is currently unknown whether this hyperleptinemia in females leads to disruptions in hypothalamic feeding circuitry as it does in males, and the precedent for major sex differences in hypothalamic outcomes after neonatal overfeeding suggests a similarity to male responses cannot be assumed for females. We, therefore, aimed to determine if neonatal overfeeding-induced hyperleptinemia would compromise hypothalamic connectivity and hypothalamic responsiveness to leptin in females as it does in males.

## Materials and Methods

### Animals

In these experiments, we used time-mated pregnant Wistar rats, obtained from the Animal Resources Centre, WA, Australia. On arrival at the RMIT University Animal Facility at day 14–16 of gestation, they were housed at 22°C, 12 h light/dark cycle (0700 hours to 1900 hours). We provided them with *ad libitum* pelleted standard rat chow and water. All procedures described here were conducted in accordance with the recommendations of the National Health and Medical Research Council Australia Code of Practice for the Care of Experimental Animals and the protocols were approved by the RMIT University Animal Ethics Committee.

### Litter Size Manipulation

As we have previously described (10–12) on P0 (the day of birth), we removed all the rat pups from their dams and randomly redistributed them to new dams in litters of 12 (control litter; CL) or 4 (small litter; SL, neonatal overfeeding). Care was taken that none of the dams received any of their own pups. Each new litter was made up of equal numbers of males and females. Excess pups were culled by rapid decapitation. As we have previously seen, SL pups were significantly heavier than CL by P7 (10–12). Experimental animals were culled at P7, P12, or P14. Only females were used in the experiments described here. Data from the males of the same litters were used in other publications (13, 27). We derived all experimental groups from three or more litters, using a maximum of two pups from the same litter for an experimental treatment to control for maternal effects (31).

### Effects of Neonatal Overfeeding on Neonatal Circulating Leptin and Triglycerides

On P7 or P14, we rapidly decapitated the animals and collected trunk blood for later assessment of plasma leptin and triglycerides. Whole blood was collected in EDTA-coated tubes, kept on ice and quickly centrifuged to separate the plasma. The plasma samples were aliquoted and stored at −20°C avoiding freeze–thaw cycles until use.

To determine leptin concentrations in our samples, we performed a standard commercial leptin ELISA, following the manufacturer’s instructions (Millipore, Ballerica, MA, USA). Intra-assay variability was 1.9–2.5% CV, inter-assay variability, 3.0–3.9% CV, and lower limit of detection, 0.04 ng/ml. All compared samples were assayed in duplicates and processed in the same assay.

To determine triglyceride concentrations in our samples, we performed a triglyceride assay (Cayman, Ann Arbor, MI, USA) according to the manufacturer’s instructions. Intra-assay coefficient of variation was 1.34%, inter-assay coefficient of variation was 3.17%, and the lower limit of detection for this assay was 0.5 mg/dl. All samples were assessed in duplicates and under the same conditions.

### Effects of Neonatal Overfeeding on Hypothalamic Gene Expression

We used quantitative real time RT-PCR (qRT-PCR) to assess whether neonatal overfeeding alters hypothalamic gene expression of the *Npy, Agrp, Pomc*, or leptin receptor (*Lepr*) in the ARC and hypothalamus from the animals described above (see Table 1 for primer details). We dissected the brains into the right ARC and left hypothalamus not containing ARC as previously described (13). These samples were immediately snap-frozen in liquid nitrogen and stored at −80°C until use. RNA was purified using QIAzol reagents and RNeasy Mini Kits (QIAGEN, Valencia, CA, USA). The RNA concentration was determined using a spectrophotometer (NanoDrop One, Thermo Scientific, Waltham, MA, USA). 1 µg RNA was transcribed to cDNA using iScript cDNA synthesis kits (QIAGEN) according to manufacturer’s instructions. A qRT-PCR Taqman Gene Expression Assay (Applied Biosystems, Mulgrave, VIC, Australia) was performed on an Applied Biosystems™ QuantStudio™ 7 Flex qPCR System instrument (Life Technologies, Carlsbad, CA, USA), and the relative quantitative measure of the target gene expression was compared with an endogenous control, β*-actin*. β*-actin* was not significantly different between any of the groups. RNA expression was determined using the double delta (*C(t)*) equation 2^−ΔΔ^*^*C(t)*^*, where threshold cycle [*C(t)*] values were the values at which fluorescence was first detected significantly above background, as previously described (13). Minus reverse-transcriptase (−RT), with omitted reverse transcriptase reactions, and no template controls (NTC), with omitted primer reactions, were run simultaneously with the samples in order to verify no genomic contamination was present. The mean *C(t)* value of our −RT test samples for β*-actin* was more than 10 cycles different from the mean *C(t)* value of our test samples, indicative of a twofold difference in the initial template amount, therefore, allowing us to assume 100% efficiency and the presence of negligible genomic DNA. NTC were undetermined [>40 C*(t)*], suggesting there was no DNA contamination. Data are presented as fold increase relative to P7 CLs. Since POMC cell bodies are found exclusively in two central nervous system nuclei, the ARC and the nucleus tractus solitarius (32, 33), we have not examined POMC gene expression in the hypothalamic tissue that did not contain the ARC. Since hypothalamic AgRP neurons are only found in the ARC, we also did not examine AgRP in the hypothalamus not containing ARC.

**Table 1:** Primer details for quantitative real-time PCR.

Primer name	NCBI reference sequence	TaqMan assay ID	Product size
*Actb*	NM_031144.2	4352340E	91
*Lepr*	NM_012596	Rn01433205_ml	94
*Npy*	NM_012614.2	Rn00561681_m1	63
*Agrp*	NM_033650.1	Rn01431703_g1	67
*Pomc*	NM_139326.2	Rn00595020_m1	92

### Neuronal Activation in Response to Exogenous Leptin

To assess if neonatal overfeeding influences the ability of the hypothalamus to respond to circulating leptin, we assessed neuronal activation in response to 3 mg/kg i.p. leptin, or equivalent volume of 0.9% sterile saline, at P12. Pups were weighed immediately before and 45 min after injection. For immunohistochemical analysis of phosphorylated signal transducer and activator of transcription 3 (pSTAT3) as a marker of leptin-induced neuronal activation, we deeply anesthetized the pups with Lethabarb (150 mg/kg pentobarbitone sodium, i.p.) 45 min after injection and perfused them transcardially with phosphate-buffered saline (PBS: 4°C, pH 7.4), followed by 4% paraformaldehyde in PBS. Brains were removed and post-fixed for 24 h in the same fixative before placing them in 20% sucrose in PBS (4°C). The forebrains were then cut into 40 µm coronal sections using a cryostat. Sections were cut into a one in five series and stored in sodium azide in PBS at 4°C until use. All our experiments were conducted between 0900 hours and 1300 hours to limit any effects of circadian rhythms in the measured parameters.

### Immunohistochemistry

Sections through the hypothalamus were immunolabelled for NPY, AgRP, POMC, and pSTAT3. We incubated one of the five series of sections from each animal in primary antibody (NPY: 1:1,000; rabbit; overnight; 4°C; Sigma-Aldrich, St. Louis, MO, USA. AgRP: 1:500; goat; 42 h; room temperature; Neuromics Inc., MN, USA. POMC: 1:5,000; rabbit; overnight; 4°C; Phoenix Pharmaceuticals, Burlingame, CA, USA. pSTAT3: 1:5,000; rabbit; overnight; 4°C; Abcam, Cambridge, UK), followed by secondary antibody (NPY: 2 h; 1:500; Alexa-fluor 488 goat anti-rabbit; Thermo Scientific, Rockford, IL, USA. AgRP: 12 h 1:200 Alexa-fluor 594 rabbit anti-goat; Thermo Scientific. POMC: 1 h; 1:500; Alexa-fluor 488 goat anti-rabbit; Thermo Scientific. pSTAT3: 1.5 h; 1:500; biotinylated anti-rabbit; Vector Laboratories, Burlingame, CA, USA). pSTAT3 sections were then incubated with avidin-biotin horseradish peroxidase (HRP) complex (ABC; 45 min; Vector Elite kit; Vector), followed by diaminobenzidine intensified with nickel to visualize the HRP activity. We stopped the reaction when the optimal contrast between the specific cellular and non-specific background labeling was observed. We air-dried the brain sections, dehydrated them in a series of alcohols, cleared them in histolene, and coverslipped. NPY, AgRP, and POMC sections were counterstained with DAPI for 15 min and mounted on slides with DAKO anti-fading solution.

The specificities of the antibodies used in our study have been previously validated by manufacturers and other researchers in pre-absorption and Western blotting experiments and further supported in our own validation experiments by incubation of experimental tissue without a primary or without a secondary antibody. Negligible positive labeling was seen in these negative controls (not shown). Positive labeling was confirmed in the brain regions of interest. More specifically, the NPY antibody (N9528) has been validated by the manufacturer and used in Ref. (34–37). The AgRP antibody (GT15023) has been validated by the manufacturer and used by Ref. (35, 38–43). The POMC antibody (H-029-30) specificity has been confirmed by Ref. (44) and used by Ref. (45–52). The pSTAT3 antibody (ab76315) has been previously validated by the manufacturer by Western blotting in HeLa cell lysate and used in the following publications (53–55).

Hypothalamic sections were assessed by an experimenter blinded to treatment groups. To visualize NPY, AgRP, and POMC labeling, photomicrographs were taken on an upright confocal laser-scanning microscope (Nikon Eclipse 90i, Tokyo, Japan) and visualized under 525/50 (NPY and POMC), 695/50 (AgRP), and 450/50 (DAPI) detection filters. ARC and PVN images were viewed under the 20× objective lens. NIS Elements Advanced Research Software (Nikon) was used to analyze fluorescence signal intensity within the ARC and PVN. Laser and detector levels were kept constant throughout the imaging. NPY, AgRP, and POMC cell fiber density were detected by the thresholding method as previously described (13). Four brain sections 120 µm apart between 2.76 and 3.48 mm caudal to bregma per animal were analyzed. POMC-positive cells in the ARC were manually counted using Image J (National Institutes of Health, Bethesda, MD, USA). For the numbers of cells positive for pSTAT3, total immunoreactive cell numbers in the ARC and ventromedial hypothalamus (VMH) were counted. Summed counts of the four sections were taken as our sampled result.

### Data Analysis

All data were analyzed using multifactorial analyses of variance (ANOVAs) with neonatal nutritional environment (CL/SL) and age (P7/14) or leptin treatment (saline/leptin) as between factors. Data were tested for homogeneity of variance and normality, using the Levene’s test for Equality of Variance and the Shapiro–Wilks test, respectively, complemented by the assessment of skewness and kurtosis. Where significant interactions were found, we then performed Tukey *post hoc* tests. Immunoreactivity results were analyzed with Student’s unpaired *t*-tests. Data are presented as the mean ± SEM. Statistical significance was assumed when *p* ≤ 0.05.

## Results

### Neonatal Overfeeding Leads to Accelerated Weight Gain

**Figure 1:**
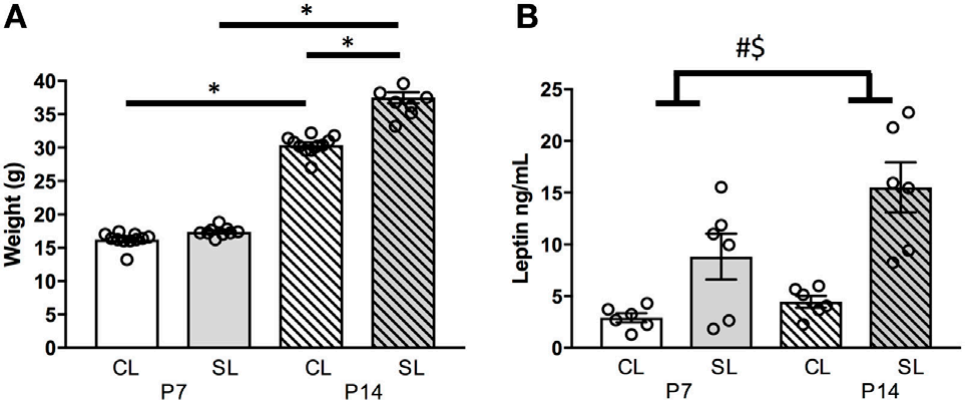
Effects of neonatal overfeeding on the neonatal leptin system. **(A)** Body weights of control (CL) and small litter (SL) rats at postnatal day (P) 7 and 14, **(B)** plasma leptin concentrations at P7 and P14. Data are mean ± SEM. *n* = 6–10 per group. *Tukey’s *post hoc*. # significant main effect of litter size. $ significant main effect of age. *p* < 0.05. Note, in **(A)**, error bars are overlapped by the data point circles.

In this cohort of rats, neonatal overfeeding led to early weight gain. Neonatally overfed (SL) rats were thus significantly heavier than controls [CL; significant age by litter size interaction: *F*_(1, 32)_ = 30.37, *p* < 0.001; *n* = 8–10; Figure 1A]. *Post hoc* analyses indicated that, by P14, the SL were significantly heavier than CL. These findings are similar to those we have previously published (10–12).

### Neonatal Overfeeding Effects on Circulating Leptin

We have previously seen that circulating leptin levels are markedly higher in neonatally overfed than in control female (and male) rats during the neonatal period, and into adulthood, and we replicated the neonatal findings here (5, 12, 13) [significant effect of litter size: *F*_(1, 20)_ = 25.35, *p* < 0.001; significant effect of age: *F*_(1, 20)_ = 6.01, *p* = 0.024, *n* = 6 per group, Figure 1B].

### Neonatal Overfeeding Effects on Neonatal Hypothalamic Feeding-Related Gene Expression

**Figure 2:**
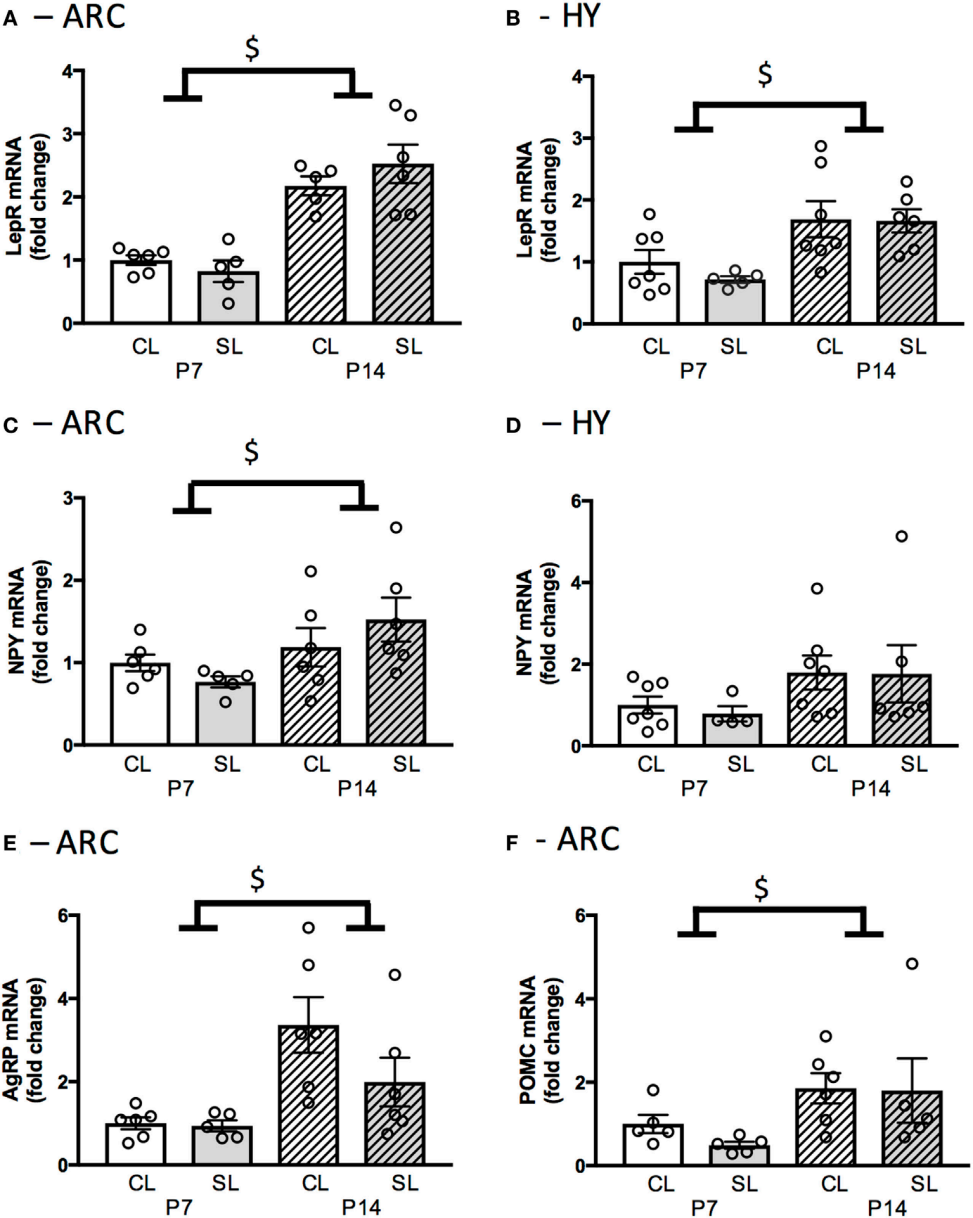
Neonatal overfeeding effects on neonatal hypothalamic feeding-related gene expression. **(A)** Leptin receptor expression in the arcuate nucleus of the hypothalamus (ARC) and **(B)** hypothalamus (HY) of control (CL) and small litter (SL) rats at postnatal day (P) 7 and P14. **(C)** Neuropeptide Y (NPY) gene expression in the ARC and **(D)** in the HY. **(E)** Agouti-related peptide (AgRP) gene expression in the ARC. **(F)** Pro-opiomelanocortin (POMC) gene expression in the ARC. Data are mean ± SEM. *n* = 5–6 per group. ^$^Significant main effect of age. *p* < 0.05.

Neonatal overfeeding did not affect hypothalamic satiety-related genes measured here. However, significant age-related changes in these genes were observed. Expression of *Lepr* mRNA in the ARC and the hypothalamus was not affected by neonatal overfeeding, but it was significantly increased at P14 compared to P7 in the ARC [significant effect of age: *F*_(1, 21)_ = 23.07, *p* < 0.001; *n* = 5–6; Figure 2A] and the hypothalamus [significant effect of age: *F*_(1, 21)_ = 13.83, *p* < 0.001; *n* = 5–7; Figure 2B] in both groups.

*Npy* mRNA expression in the ARC was not affected by neonatal overfeeding, but it was significantly increased at P14 compared to P7 [significant effect of age: *F*_(1, 19)_ = 5.84, *p* = 0.026; *n* = 5–6; Figure 2C]. In the hypothalamus, neonatal overfeeding had no effect on *Npy* expression (Figure 2D). *Agrp* mRNA expression in the ARC was not affected by neonatal overfeeding; however, it was significantly increased at P14 compared to P7 [significant effect of age: *F*_(1, 19)_ = 12.86, *p* = 0.002; *n* = 5–6; Figure 2E]. Neonatal overfeeding also did not affect *Pomc* mRNA expression in the ARC. However, again, there was a significant increase of *Pomc* mRNA in P14 ARC compared to P7 [significant effect of age: *F*_(1, 17)_ = 6.16, *p* = 0.024; *n* = 5–6; Figure 2F].

### Neonatal Overfeeding Effects on Neonatal Hypothalamic NPY, AgRP, POMC

**Figure 3:**
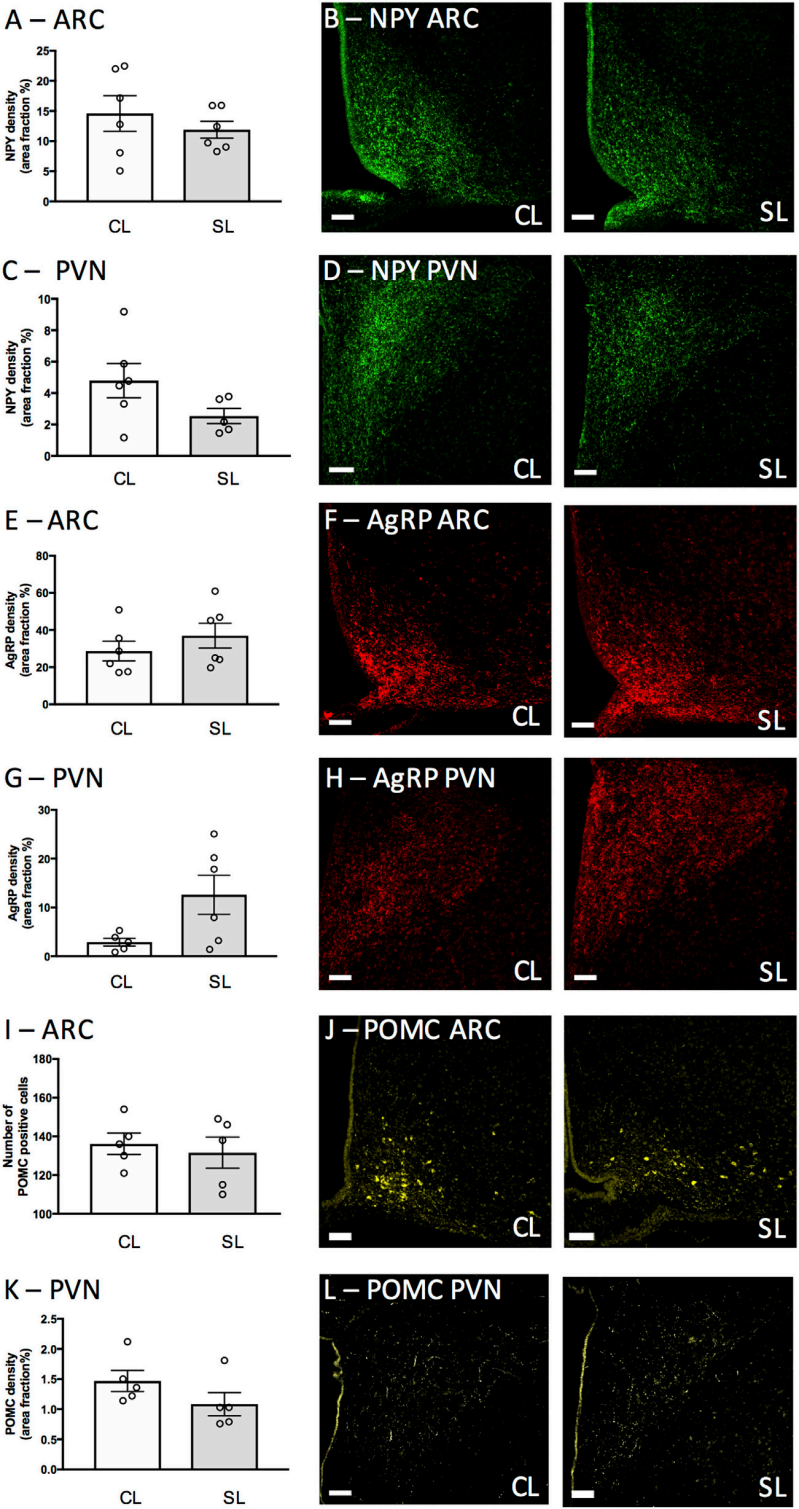
Neonatal overfeeding effects on neonatal hypothalamic circuitry. **(A,B)** neuropeptide Y (NPY) labeling in the arcuate nucleus of the hypothalamus (ARC) and **(C,D)** paraventricular nucleus of the hypothalamus (PVN) of control (CL) and small litter (SL) rats at postnatal day (P) 12. **(E,F)** Agouti-related peptide (AgRP) labeling in the ARC and **(G,H)** PVN. **(I,J)** Proopiomelanocortin (POMC) positive cells in the ARC. **(K,L)** POMC labeling in the PVN. Representative photomicrographs showing NPY labeling in the **(B)** ARC and **(D)** PVN, AgRP labeling in the **(F)** ARC, and **(H)** PVN, POMC labeling in the **(J)** ARC and **(L)** PVN. Scale bars = 100 µm. Data are mean ± SEM. *n* = 5–6 per group.

To determine if the elevated leptin in neonatally overfed females was associated with a disruption of hypothalamic NPY, AgRP, and POMC as previously described in males (13), we examined NPY fibers (ARC: Figures 3A,B, PVN: Figures 3C,D), AgRP fibers (ARC: Figures 3E,F, PVN: Figures 3G,H), and POMC-positive cells in the ARC (Figures 3I,J) and POMC fibers in the PVN (Figures 3K,L) at P12. Neonatal overfeeding did not affect NPY, AgRP, and POMC immunoreactive fibers or POMC immunoreactive cells in these regions (*n* = 5–6 per group).

### Neonatal Overfeeding Effects on Neonatal Hypothalamic Responses to Leptin

**Figure 4:**
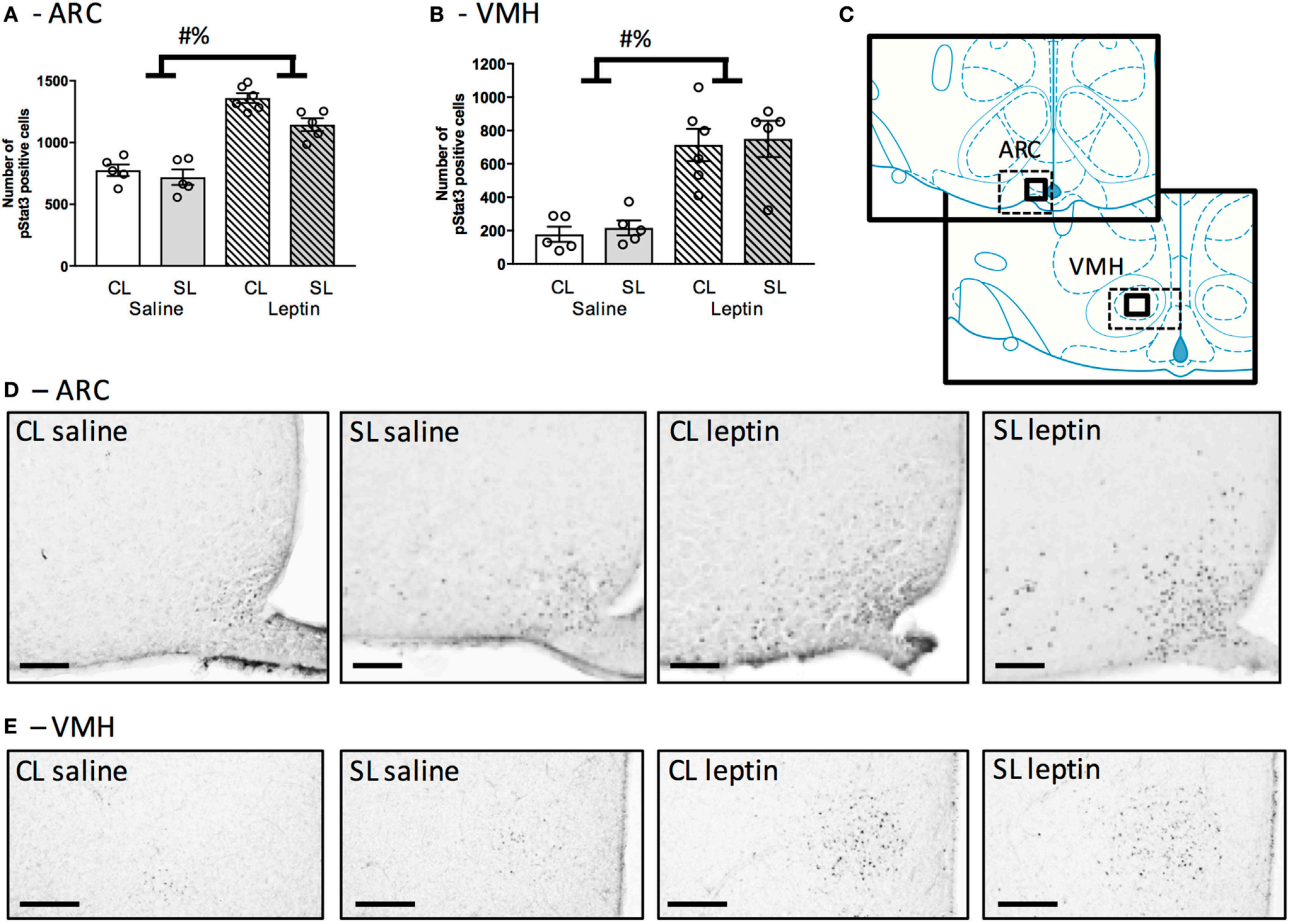
Neonatal overfeeding effects on neonatal hypothalamic responses to leptin. **(A)** Arcuate nucleus of the hypothalamus (ARC) and **(B)** ventromedial hypothalamus (VMH) neuronal activation in response to leptin injection at postnatal day (P) 12 in control (CL) and small litter (SL) rats as assessed by numbers of signal transducer and activator of transcription (pSTAT3) positive cells. The sum of cell counts in four sections was plotted. **(C)** Schematic diagram adapted from Paxinos and Watson illustrating the regions of interest. Thick-line boxes are representative of analyzed regions, dotted-line boxes are representative of the photomicrographs. Representative photomicrographs of pSTAT3 after a saline or leptin injection in the **(D)** ARC (scale bars = 100 µm) and **(E)** VMH (scale bars = 200 µm). Data are mean ± SEM. *n* = 5–6 per group. # significant main effect of litter size. % significant main effect of leptin. *p* < 0.05.

We next tested if neonatal overfeeding alters the ability of the neonatal hypothalamus to respond to a leptin signal by giving the pups a single injection of leptin, or saline, on P12 and measuring weight changes as well as hypothalamic pSTAT3 expression. There were no differences in the weight gain after 45 min in any of the groups (data not shown). Leptin stimulated an increase in the number of pStat3 positive cells in the ARC, as expected [*F*_(1, 17)_ = 101.30, *p* < 0.001; *n* = 5–6]. There was also a significant main effect of litter size [*F*_(1, 17)_ = 7.36, *p* = 0.015; Figures 4A,C,D], but no interaction. In the VMH, leptin injection also induced a significant increase in pSTAT3-positive cells [main effect of leptin: *F*_(1, 17)_ = 42.84, *p* < 0.001; *n* = 5–6; Figures 4B,C,E].

### Neonatal Overfeeding Effects on Neonatal Circulating Triglycerides

**Figure 5:**
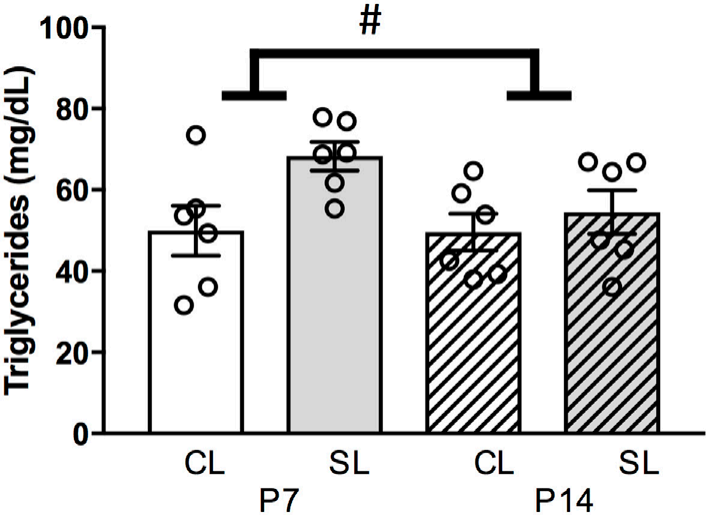
Neonatal overfeeding effects on neonatal circulating triglycerides. Plasma triglyceride concentration at postnatal day (P)7 and P14 in control (CL) and small litter (SL) rats. Data are mean ± SEM. *n* = 6 per group. # significant main effect of litter size. *p* < 0.05.

Since neonatal overfeeding had minimal effects on hypothalamic feeding circuitry in female rats despite inducing pronounced neonatal hyperleptinemia, we next examined circulating triglyceride levels. Triglycerides have been shown to mediate leptin transport to the brain (56). We, therefore, hypothesized neonatal overfeeding might lead to elevated circulating triglycerides that could reduce leptin transport to the brain and thus reduce the effects of hyperleptinemia. In this regard, neonatal overfeeding led to an increase in circulating triglyceride levels [significant effect of litter size: *F*_(1, 20)_ = 5.43, *p* = 0.030; *n* = 6 per group, Figure 5], without an effect of age.

## Discussion

The early life nutritional environment plays a crucial role in metabolism and neurodevelopment. Here, for the first time, we show that neonatal overfeeding in females, despite hyperleptinemia and a corresponding increased body weight, does not affect NPY, AgRP, and POMC mRNA or protein in the hypothalamic circuitry responsible for feeding and metabolic control. These observations are different from findings previously shown in neonatally overfed males, where overfeeding leading to hyperleptinemia and increased body weight are associated with short-term disruption of hypothalamic neuronal wiring responsible for metabolic regulation (13, 26).

Naturally occurring high circulating levels of leptin are seen at approximately P4 to P16 in mice (57) and P4 to P14 in rats (58) with a peak at P10 (59), they then normalize toward adult levels after weaning (21). Such an increase in leptin levels is not associated with acute changes in food intake, but is reflective of leptin’s neurodevelopmental role in stimulating the growth of hypothalamic connections between the ARC and other hypothalamic regions that are ultimately responsible for controlling energy balance (21). Disruptions to this leptin surge can permanently impact upon the development of these hypothalamic connections and lead to aberrant feeding behavior and metabolism throughout life (20, 22). For example, leptin-deficient (ob/ob) male mice have a lack of innervation developing from the ARC toward the PVN and other hypothalamic regions (20), deficits that can be repaired by restoration of normal neonatal leptin levels (20). Being suckled in a small litter during the first 3 weeks of life in mice and rats can similarly disrupt hypothalamic circuitry. Thus, early life overfeeding increases body weight, fat mass, and circulating leptin in comparison to the normally fed. In males, this hyperleptinemia is associated with a disruption in NPY and AgRP fibers and leads to an obese phenotype that is maintained throughout life (12, 14, 60, 61).

It is important to note that most of the current knowledge in regards to the effects of postnatal overfeeding on hypothalamic neuronal development derives from observations in male rodents. However, changes in the neonatal leptin availability as a result of overfeeding appear to induce sex-dependent effects on the development of hypothalamic neuronal connectivity. We observe here that in females, similarly to males, neonatal overfeeding is associated with exacerbated hyperleptinemia during the neonatal leptin surge period, and this is followed by an increase in body weight. However, neonatally overfed females are still responsive to leptin and, importantly, there is no effect of female neonatal overfeeding on the mRNA levels of the *Lepr*, or in the changes in the gene or protein expression of NPY, AgRP, or POMC in the hypothalamus. The ARC of small litter animals showed decreased sensitivity to leptin, although there was no interaction between litter size and leptin. However, the magnitude of this difference is small, of the order of 15% of the response in the case of the leptin treated. We also do not see this difference in the VMH. We observe here that neonatally overfed females have increased circulating triglyceride levels relative to controls. It is thus possible that the hyperleptinemia in females is compensated for by elevated blood triglycerides restricting leptin’s access to the brain in the neonatally overfed (56), which may also account for the minor reduction in the number of pSTAT3-positive cells in the ARC of these animals. However, this observation does not explain why males are not also resistant to the effects of excess leptin, since we would expect males to also have increased circulating triglyceride levels when neonatally overfed. There is some evidence of sex differences in triglyceride levels in adults after an early food restriction. For instance, there are elevated triglycerides in perinatally food-restricted females relative to males (62), while other perinatal insults, such as maternal deprivation (63), have been shown to increase circulating triglycerides in adult males, but decrease them in females. However, no direct sex comparison has yet been made during the neonatal phase.

We have previously seen sex differences in the way that neonatally overfed rats control energy expenditure. Female rats that are overfed as neonates remain fat into adulthood and they do this not by overeating, but by reducing energy expenditure, probably due to reduced activation of brown adipose tissue (BAT) during the first half of the dark phase (12). BAT is responsible for the conversion of energy from food into heat primarily *via* uncoupling protein (64) and neonatally overfed females are unable to optimally convert BAT into energy and show reduced energy consumption at the juvenile stage, with normalization of function in adulthood. This disruption of BAT function at the juvenile stage is not evident in the neonatally overfed males (12). BAT thermogenesis is reduced in neonatally overfed females until P30 but, by adulthood, BAT thermogenesis normalizes to controls levels (12), a possible explanation of this is how these females retain an elevated body weight without any changes in their hypothalamic feeding networks.

Another potential reason for these sex-dependent effects of neonatal overfeeding may lie in the organization of sexually dimorphic neural pathways. Sex steroid hormones such as estrogen and testosterone, in combination with neurotrophins, are able to regulate formation of sexually dimorphic circuits by affecting axonal guidance and synaptogenesis [reviewed in Ref. (65)]. Significant hormonal changes in the central nervous system in rodents happen partly due to the gonadal steroid hormones during sexual differentiation (66). For instance, sexually dimorphic nucleus, a cluster of cells in the preoptic area of the hypothalamus responsible for controlling sexual behavior by affecting sex hormones such as testosterone and estrogens in rats, develops from as early as P1. By P8, this region is almost twice the size in males as that of females [reviewed in Ref. (67)]. Conversely, the anteroventral periventricular nucleus, a region of the hypothalamus responsible for the pulsatile release of gonadotropin releasing hormone, covers a larger area and consists of a larger number of neurons in females than in males [reviewed in Ref. (65)]. Testosterone and estrogens influence energy homeostasis at least partially *via* hormonal receptors, which are colocalized with hunger or satiety neuropeptides located in the hypothalamus [reviewed in Ref. (68)], suggesting that sexual dimorphism affects the development of hypothalamic regions that control energy balance and may thus also be reflected in the differential effects of neonatal overfeeding on hypothalamic appetite-regulatory circuitry.

Overall, our results suggest females overfed during early life, despite being hyperleptinemic and experiencing an obesogenic phenotype, are not acutely affected in their central neuronal connectivity responsible for metabolic control. These results contrast with our and others’ previous findings in males. These findings are potentially reflective of differences in how females and males adapt to early life environmental dietary challenges. Our work further highlights that it is important not to assume female physiology from male data and that different physiological mechanisms may lead to a similar phenotypic outcome, in this case, excess body weight.

## Ethics Statement

All procedures described here were conducted in accordance with the recommendations of the National Health and Medical Research Council Australia Code of Practice for the Care of Experimental Animals and the protocols were approved by the RMIT University Animal Ethics Committee.

## Author Contributions

SS conceived of and designed the work. IZ, LS, T-XN, K-YY, SL, AK, SS (i.e., all authors) made substantial contributions to the acquisition, analysis, and/or interpretation of data for the work. IZ, LS, and SS drafted the work. All authors critically revised it for important intellectual content. All authors give final approval of the version to be published. All authors agree to be accountable for all aspects of the work in ensuring that questions related to the accuracy or integrity of any part of the work are appropriately investigated and resolved.

## Conflict of Interest Statement

The authors declare that the research was conducted in the absence of any commercial or financial relationships that could be construed as a potential conflict of interest.
